# Morphogenesis of the femur at different stages of normal human development

**DOI:** 10.1371/journal.pone.0221569

**Published:** 2019-08-23

**Authors:** Yuko Suzuki, Jun Matsubayashi, Xiang Ji, Shigehito Yamada, Akio Yoneyama, Hirohiko Imai, Tetsuya Matsuda, Tomoki Aoyama, Tetsuya Takakuwa

**Affiliations:** 1 Human Health Science, Graduate School of Medicine, Kyoto University, Kyoto, Japan; 2 Congenital Anomaly Research Center, Graduate School of Medicine, Kyoto University, Kyoto, Japan; 3 SAGA Light Source, Saga, Japan; 4 Department of Systems Science, Graduate School of Informatics, Kyoto University, Kyoto, Japan; Ohio State University, UNITED STATES

## Abstract

The present study aimed to better characterize the morphogenesis of the femur from the embryonic to the early fetal periods. Sixty-two human fetal specimens (crown–rump length [CRL] range: 11.4–185 mm) from the Kyoto Collection were used for this study. The morphogenesis and internal differentiation process of the femur were analyzed in 3D using phase-contrast X-ray computed tomography and magnetic resonance imaging. The cartilaginous femur was first observed at Carnegie stage 18. Major anatomical landmarks were formed prior to the initiation of ossification at the center of the diaphysis (CRL, 40 mm), as described by Bardeen. The region with very high signal intensity (phase 5 according to Streeter’s classification; i.e., area described as cartilage disintegration) emerged at the center of the diaphysis, which split the region with slightly low signal intensity (phase 4; i.e., cartilage cells of maximum size) in fetuses with a CRL of 40.0 mm. The phase 4 and phase 5 regions became confined to the metaphysis, which might become the epiphyseal cartilage plate. Femur length and ossified shaft length (OSL) showed a strong positive correlation with CRL. The OSL-to-femur length ratio rapidly increased in fetuses with CRL between 40 and 75 mm, which became moderately increased in fetuses with a CRL of ≥75 mm. Cartilage canal invasion occurred earlier at the proximal epiphysis (CRL, 62 mm) than at the distal epiphysis (CRL, 75 mm). Morphometry and Procrustes analysis indicated that changes in the femur shape after ossification were limited, which were mainly detected at the time of initial ossification and shortly after that. In contrast, femoral neck anteversion and torsion of the femoral head continuously changed during the fetal period. Our data could aid in understanding the morphogenesis of the femur and in differentiating normal and abnormal development during the early fetal period.

## Introduction

The femur is a long bone that develops via endochondral ossification. In particular, the human femur first appears as mesenchymal condensation between Carnegie stage (CS) 16 and CS17. Chondrification occurs between CS17 and CS18 and subsequently proceeds to endochondral ossification between CS22 and CS23 [1–3]. Most previous studies have been focused on the differentiation processes after the appearance of a primary ossification center at the femoral diaphysis [4–6]. Gardner and Gray (1970) described that the invasion and destruction of calcified cartilage cells occurred in fetuses with a crown–rump length (CRL) of 37 mm and that endochondral ossification and endochondral trabecular formation were prominent in fetuses with a CRL of 57 mm [4]. This process rapidly progresses toward each end of the femur. Recent evaluation of the role of chondrocytes in endochondral differentiation has supported the concept of transdifferentiation during endochondral ossification [7,8]. Specifically, suspected cartilage fragmentation may be irregularly shaped residual matrix present during cartilage-to-osteoblast transdifferentiation. At 15–20 weeks of gestation, the diaphysis exhibits cancellous bone formation with scattered trabeculae [5]. Cartilage canals are minute tunnels containing blood vessels, which are the forerunners of the epiphyseal nutrient arteries and veins and their rami in bony epiphyses. These formations precede the formation of a secondary ossification center and are crucial for the establishment of the secondary ossification center observed after birth [9]. Cartilage canal formation is observed initially in the proximal epiphysis of fetuses with a CRL of 57 mm and subsequently in the distal epiphysis of fetuses with a CRL of 61 mm [4].

The cartilage structure influences bone structure formation, as ossification occurs as if the cartilage structure is the blue print replaced by the bone structure. Identifying how the normal cartilage is formed before ossification is essential. Nevertheless, the initial morphogenesis of the femur before ossification (chondrogenic stage) during the embryonic period has not been fully described, except for in limited classical studies [10,11]. Furthermore, how the morphological features of the cartilage structure may be replaced by those of the bone structure has not been fully shown, because previous studies had mainly examined two-dimensional (2-D) sections using histology and radiographs or gross view after dissection and these lacked precise quantitative information about the three-dimensional (3-D) formation of the femur. For example, previous studies had reported angle measurements such as femoral neck anteversion (∠FNA), femoral neck shaft angle (∠FNS), and obliquity of the shaft (∠OBS) during the fetal period. Such measurements may be useful for indicating changes in the shape of the femur, although data remain limited and controversial [12–14].

The present study aimed to further characterize femur morphogenesis by examining successive stages from cartilage condensation to endochondral ossification. Therefore, the morphogenesis of the femur in the chondrogenic stage and the subsequent endochondral ossification were analyzed in 3-D using phase-contrast X-ray computed tomography (PCX-CT) and magnetic resonance (MR) images. In addition, the internal differentiation process, including ossification and cavity formation at the diaphysis and cartilage canal formation at the epiphysis, was analyzed. Procrustes analysis was performed to distinguish the change in shape from the change in size according to growth.

## Materials and methods

### Human fetal specimens

A total of 62 human embryo and fetal specimens (13 embryo specimens from CS18 to CS23 [CRL range: 11.4–33.5 mm] and 49 fetal specimens [CRL range: 37.2–185 mm]) from the Kyoto Collection at the Congenital Anomaly Research Center of Kyoto University [15–17] were used for this study. Most specimens stored at the Kyoto Collection were acquired when pregnancy was terminated for socioeconomic reasons under the Maternity Protection Law of Japan. Parents provided their verbal informed consent to have the specimens deposited in the collection, and participant consent was documented in each medical record. Written consent was not obtained from all parents. Samples were collected from 1963 to 1995 in accordance with the regulations relevant at each time. Some of these specimens were undamaged and well-preserved fetuses. The aborted fetal specimens were brought to the laboratory, measured, examined, and staged using the criteria proposed by O’Rahilly and Müller [1].

The ethics committee of Kyoto University Faculty and Graduate School of Medicine approved this study, which used human embryo and fetal specimens (E986, R0316).

### Image acquisition

The image acquisition parameters for 3-D PCX-CT had been described previously [18]. Briefly, specimens were visualized using a phase-contrast imaging system fitted with a crystal X-ray interferometer [19]. The system was set up at the vertical wiggler beamline (PF BL-14C) of the Photon Factory in Tsukuba, Japan. The white synchrotron radiation emitted from the wiggler was monochromated by a Si(220) double-crystal monochromator, horizontally magnified by an asymmetric crystal, and inputted into the imaging system. The generated interference patterns were detected by a large-area X-ray imager, which consisted of a 30-μm scintillator, relay lens system, and water-cooled charge-coupled device camera (36 × 36 mm field of view, 2048 × 2048 pixels, 18 × 18 μm each) [20]. The X-ray energy was tuned at 17.8 keV, and an exposure time of 3 s was used to obtain one interference pattern. The average intensity was approximately 300 counts/pixel, which allowed for high-resolution observations within a reasonable measurement time.

MR images were acquired using a 7-T MR system (BioSpec 70/20 USR; Bruker BioSpin MRI GmbH, Ettlingen, Germany) and a 3-T MR system (MAGNETOM Prisma; Siemens Healthineers, Erlangen, Germany). The 7-T MR system was equipped with ^1^H quadrature transmit–receive volume coils with diameters of 35 and 72 mm (T9988 and T9562; Bruker BioSpin MRI GmbH, Ettlingen, Germany) [21]. The 3-D T1-weighted images were acquired using a fast low-angle shot pulse sequence with the following parameters: repetition time, 30 ms; echo time, 4.037–6.177 ms; flip angle, 40°; field of view, 22.5 × 15.0 × 15.0 to 42.0 × 28.0 × 28.0 μm^3^; matrix size, 636 × 424 × 424 to 768 × 512 × 512; and isotropic spatial resolution, 35.4–54.7 μm^3^.

PCX-CT was used to acquire 3-D images of specimens between CS18 and CS22, whereas MRI was used for specimens at CS23 and later stages. The 7-T MR system was used to acquire 3-D images of specimens from fetuses with CRL ranging from 22 to 112 mm and lower body specimens from fetuses with CRL of 135 mm. In contrast, the 3-T MR system was used for specimens from fetuses with CRL greater than 116 mm. The image acquisition method was selected based on specimen resolution and volume. In other words, PCX-CT was used to acquire images at a higher resolution than what could be obtained using MRI. However, PCX-CT could not be used to acquire images of specimens with large volumes. Thus, CS22 samples represented the upper limit with respect to the size of specimens that could be examined using PCX-CT.

**Fig 1:**
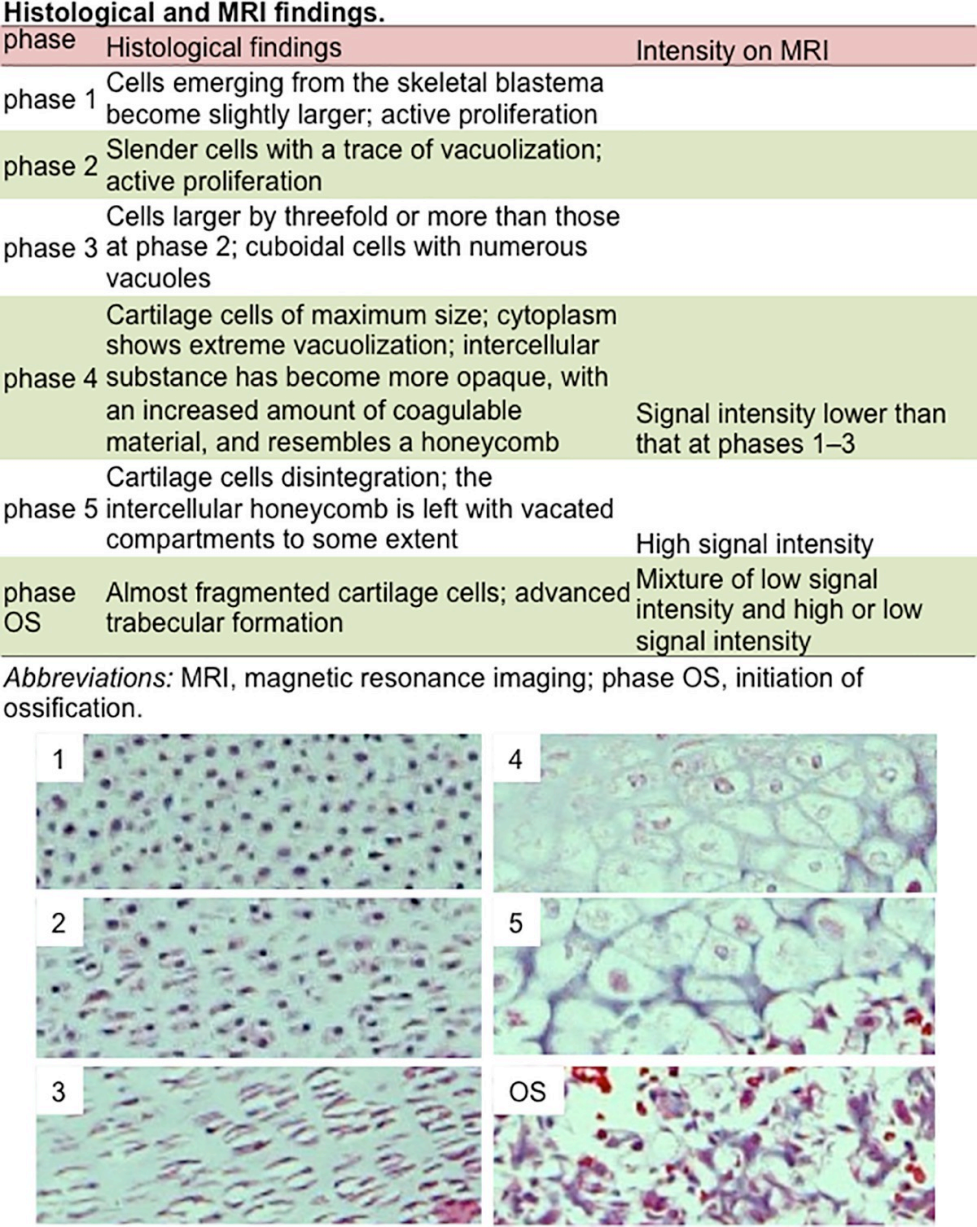
Comparison between histological and MRI findings. Endochondral ossification was assessed according to modified Streeter’s classification (1949) [22]. Representative histology was shown below. 1: Phase 1; 2: phase 2; 3: phase 3; 4: phase 4; 5: phase 5; OS: phase OS.

Endochondral ossification was assessed according to modified Streeter’s classification (1949), which was based on the histological observation of the humerus [22] (Fig 1; see also S1 Supplementary Experiments). Briefly, phases of endochondral ossification (phases 1–3, phase 4, and phase 5 according to Streeter’s classification and initiation of ossification [phase OS]) could be discerned by signal intensity and its distribution on MR images. Ossified regions (phase OS) exhibited low signal intensity or a mixture of low and high signal intensity, whereas phase 5 regions showed high signal intensity. Phase 4 regions showed low signal intensity; however, the border at the epiphysis from phases 1 to 3 was ambiguous.

### Image data analysis

PCX-CT and MRI data from selected specimens were precisely analyzed using serial 2-D images and reconstructed 3-D images. The 3-D images of the femur and ossified shaft were manually reconstructed using Amira software version 5.5.0 (Visage Imaging GmbH, Berlin, Germany).

### Morphometry

**Fig 2:**
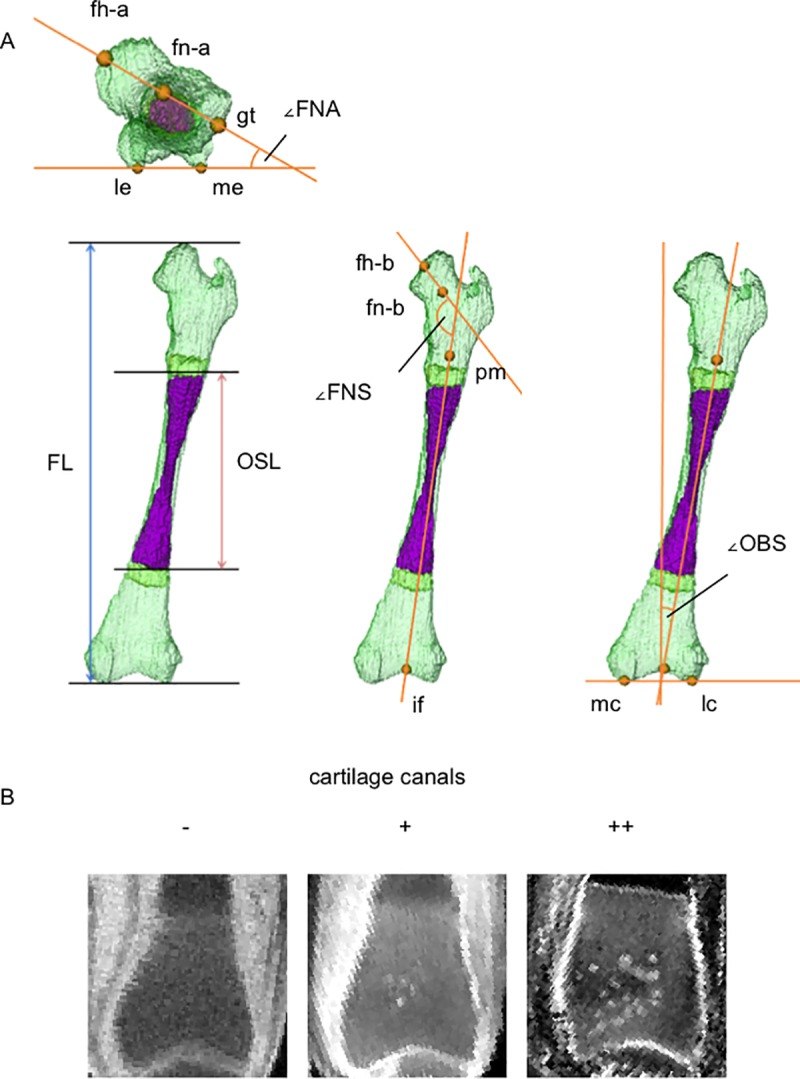
Morphometry of the femur and evaluation of cartilage canals at the epiphysis. (A) Morphometry of the femur. Method for the measurement of femur length, ossified shaft length (OSL), femoral neck anteversion (∠FNA), femoral neck shaft angle (∠FNS), and obliquity of the shaft (∠OBS). fh-a: midpoint of the femoral head as seen from above; fh-b: midpoint of the femoral head as seen from the back; fn-a: midpoint of the femoral neck as seen from above; fn-b: midpoint of the femoral neck as seen from the back; gt: midpoint of the greater trochanter as seen from above; if: intercondylar fossa as seen from the back; lc: bottom point of the lateral condyle; le: most lateral point of the lateral epicondyle as seen from above; mc: bottom point of the medial condyle; me: most lateral point of the medial epicondyle as seen from above; pm: midpoint of the proximal metaphysis just below the lesser trochanter as seen from the back. (B) Evaluation of cartilage canals at the epiphysis. Cartilage canal formation was evaluated by counting the number of cartilage canals and dividing them into three stages (-: 0, +: 1–4, ++: >5).

Femur length, ossified shaft length (OSL), ∠FNA, ∠FNS, and ∠OBS were measured to evaluate the growth and differentiation of the femur (Fig 2A). OSL was determined from MR images of fetuses with a CRL of ≥40.0 mm. ∠FNA was defined as the angle formed by the intersection of the plane passing through the lateral and medial condyles and the plane passing through the midpoint of the femoral head, femoral neck, and greater trochanter. ∠FNS was defined as the angle formed by the intersection of the femoral shaft axis and the femoral neck axis. ∠OBS was defined as the angle formed by the femoral shaft axis and the plane perpendicular to the segment of the lateral and medial condyles. The landmarks were confirmed by two of the authors (YS and TT).

### Evaluation of cartilage canal formation

Cartilage canals were observed on 7-T MR images of fetuses with CRL between 37.2 and 112 mm (n = 32). The reference longitudinal plane passing through the midpoint of the femoral head, femoral neck, and greater trochanter was defined. Cartilage canal invasion was observed on planes parallel to the reference plane at the proximal epiphysis. Cartilage canal formation was evaluated by counting the number of cartilage canals in the femoral neck, fovea, and trochanteric fossa and by dividing them into three stages (-: not detected, +: 1–4, ++: >5) (Fig 2B). Similarly, the reference horizontal plane for the distal metaphysis was defined, and cartilage canal invasion was observed on planes parallel to the reference plane at the distal epiphysis. Cartilage canal formation was evaluated by counting the number of cartilage canals in the intercondylar fossa and epicondyle and by dividing them into three stages (-: not detected, +: 1–4, ++: >5).

### Procrustes analysis

Thirteen landmarks and three semi-landmarks were defined, as shown in Table 1. Thirteen semi-landmarks were located on the femoral head (FH13), lateral condyle (LC13), and medial condyle (MC13) in order to describe the external curvature of each individual. Curves consisted of two landmark control points and eleven automatically generated semi-landmarks. Individual semi-landmarks were not determined by anatomical feature but by mathematical calculation so as to locate them at equal interval on the external curvature. The 3-D coordinates of each landmark and semi-landmark were obtained using Checkpoint (Stratovan Corporation, Davis, CA, USA) and were subjected to Procrustes analysis using MATLAB R2018b (MathWorks, Natick, MA, USA). Procrustes analysis was performed to ensure that landmark coordinates were translated, scaled, and rotated to the best superimposition. The resultant landmark coordinates were referred to as Procrustes shape coordinates, which were used for both the proximal and distal epiphyses. Changes in the position of the landmarks were observed with each Procrustes shape coordinate. The size of each specimen was represented as the centroid size, which was calculated as the square root of the sum of squared distances from the centroid to each landmark.

**Table 1:** Landmarks and semi-landmarks for Procrustes analysis.

	Name	Description
Landmarks		
	FH-f	Center of the femoral head fovea
	GT-l	Most lateral point of the greater trochanter
	GT-t	Top of the greater trochanter
	IF	Center of the intercondylar fossa
	LC-b	Bottom point of the lateral condyle
	LC-p	Most posterior point of the lateral condyle
	LE	Most lateral point of the lateral epicondyle
	LT-b	Bottom end of the lesser trochanter
	LT-t	Top of the lesser trochanter
	LT-u	Upper end of the lesser trochanter
	MC-b	Bottom point of the medial condyle
	MC-p	Most posterior point of the medial condyle
	ME	Most lateral point of the medial epicondyle
Semi-landmarks		
	FH13	13 semi-landmarks from the upper end to the lower end of the femoral head along the plane passing through the midpoint of the femoral head, femoral neck, and greater trochanter
	LC13	13 semi-landmarks along the roundness of the lateral condyle from the upper end to the opposite side
	MC13	13 semi-landmarks along the roundness of the medial condyle from the upper end to the opposite side

### Statistical analysis

Wilcoxon signed-rank test was used to examine the laterality of all three angles (i.e., ∠FNA, ∠FNS, ∠OBS). Statistical significance was set at p < 0.05. Statistical tests were run on JMP software version 14 (SAS Institute, Tokyo, Japan).

## Results

### Morphogenesis of the femur during the embryonic period

**Fig 3:**
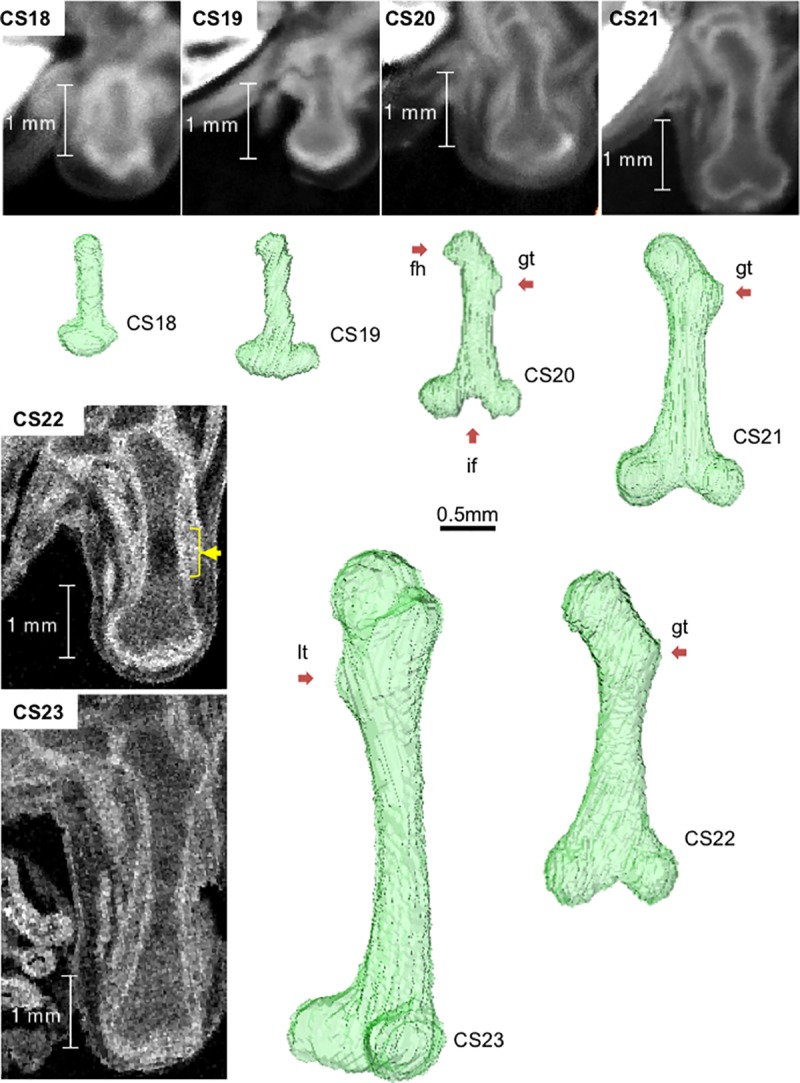
Femur development between CS18 and CS23 (before ossification). Representative longitudinal section images and 3-D reconstructed images. Longitudinal section images were acquired using PCX-CT between CS18 and CS21 and 7-T MR imaging between CS22 and CS23. fh: femoral head; gt: greater trochanter; If: intercondylar fossa; lt: lesser trochanter; yellow arrow: phase 4 according to Streeter’s classification. See also S1, S2, S3, S4, S5 and S6 Movies.

The cartilaginous femur was first observed in specimens at CS18 using PCX-CT. Specifically, the contour was detected with high signal intensity, whereas the internal portion was observed with slightly lower signal intensity (Fig 3). The 3-D reconstruction of the femur indicated a “club shape,” as the distal epiphysis was not divided into the lateral and medial condyles at CS18 (S1 Movie).

The formed intercondylar fossa divided the distal epiphysis into the lateral and medial condyles of the femur at CS20 (S3 Movie). As for the proximal epiphysis, the femoral head and greater trochanter began to be clearly discernible after CS20. The signal intensity became low at the center of the diaphysis at around CS21 on PCX-CT (yellow arrow in Fig 3). The region was detected on MR images at CS22 (yellow arrow in Fig 3). This region spread toward both epiphyses until the end of the embryonic period (CS23). The region with low signal intensity corresponded to phase 4 according to Streeter’s classification (S1 Supplementary Experiments). The femoral neck was narrow, and the lesser and greater trochanters became recognizable at CS23 (S6 Movie). The femoral head fovea was not detected. All femurs developed from cartilage tissues (phases 1–4 according to Streeter’s classification) and showed no ossification until the end of the embryonic period (CS23).

### Morphogenesis of the femur during the fetal period (CRL of 33.5–185 mm)

#### PCX-CT and MR cross-sectional images

**Fig 4:**
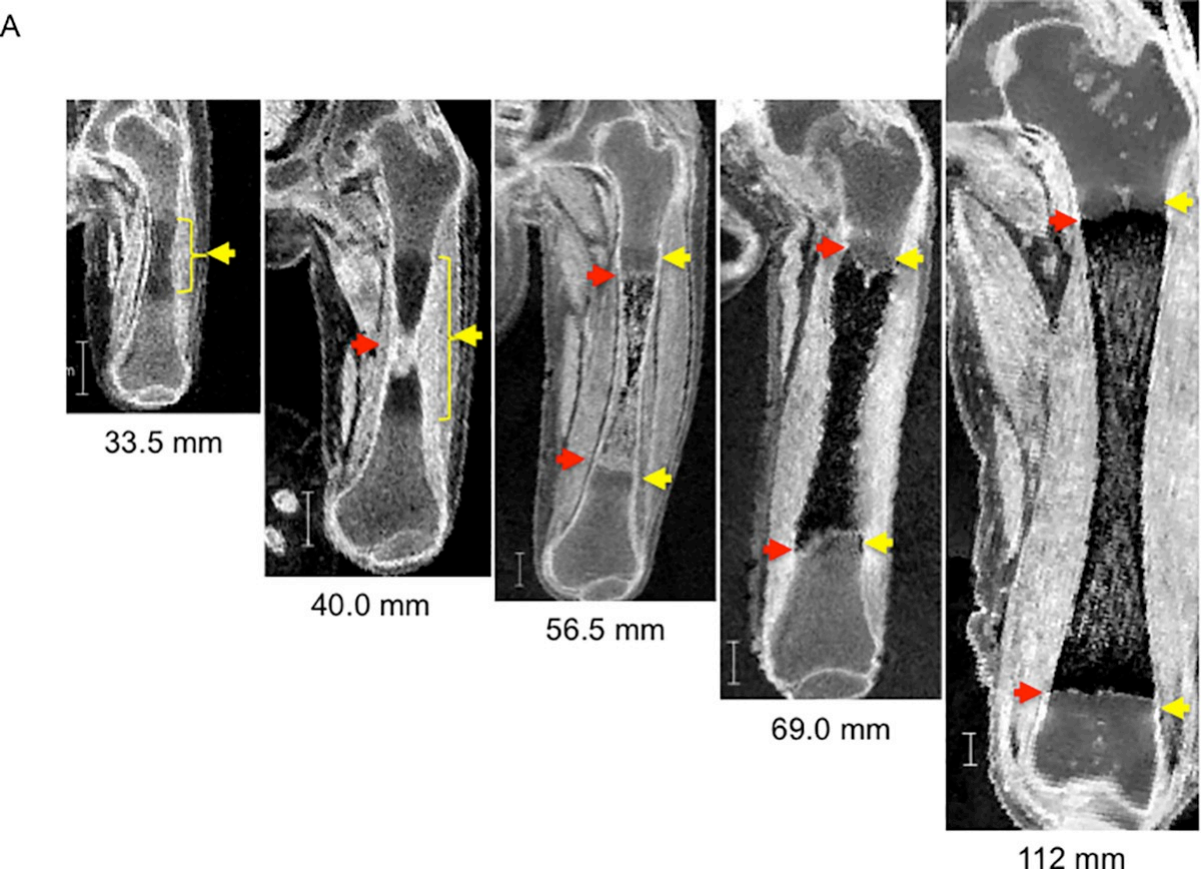
Representative cross-sectional view of the right femur during the early fetal period on 7-T MR image. The crown–rump length (CRL) of each fetus is indicated below the images. Yellow arrow: phase 4; red arrow: phase 5 according to Streeter’s classification. The region between the yellow arrows at the diaphysis corresponds to phase OS. The region outside the yellow arrows corresponds to phases 1–3.

MRI data revealed 3-D morphogenesis and internal dynamic structural changes during the embryonic and fetal periods (Fig 4). The center of the femoral diaphysis was of low signal intensity in fetuses with a CRL of 33.5 mm (4 in Fig 4). The border of the region was ill-defined, which might correspond to phase 4 for cartilage tissues observed before ossification.

The region with very high signal intensity emerged at the center of the diaphysis, which split the region with slightly low signal intensity (phase 4) in fetuses with a CRL of 40.0 mm. The high signal intensity might have resulted from the influx of blood flow and degeneration of cartilage cells, which corresponded to phase 5 and phase OS (S1 Fig). The high signal intensity in the region changed to a mixture of low and high signal intensity (netlike pattern) in fetuses with a CRL of 56.5 mm. The phase 4 (low signal intensity) and phase 5 (high signal intensity) regions became confined to the metaphysis (S2 Fig). Almost all diaphyses showed low signal intensity in fetuses with CRL ranging from 69.0 to 103 mm (S3 Fig). The border of the metaphysis became sharp with high signal intensity band (phase 5); the phase 5 region as well as the vague region with low signal intensity (phase 4) might become the epiphyseal cartilage plate. In comparison, the diaphysis showed a mixture of high and low signal intensity in fetuses with a CRL of 112 mm. All borders of the femur were recognized as regions with high signal intensity, and the periosteal color was not discernible from the border of the epiphysis on MRI.

#### 3-D reconstruction

**Fig 5:**
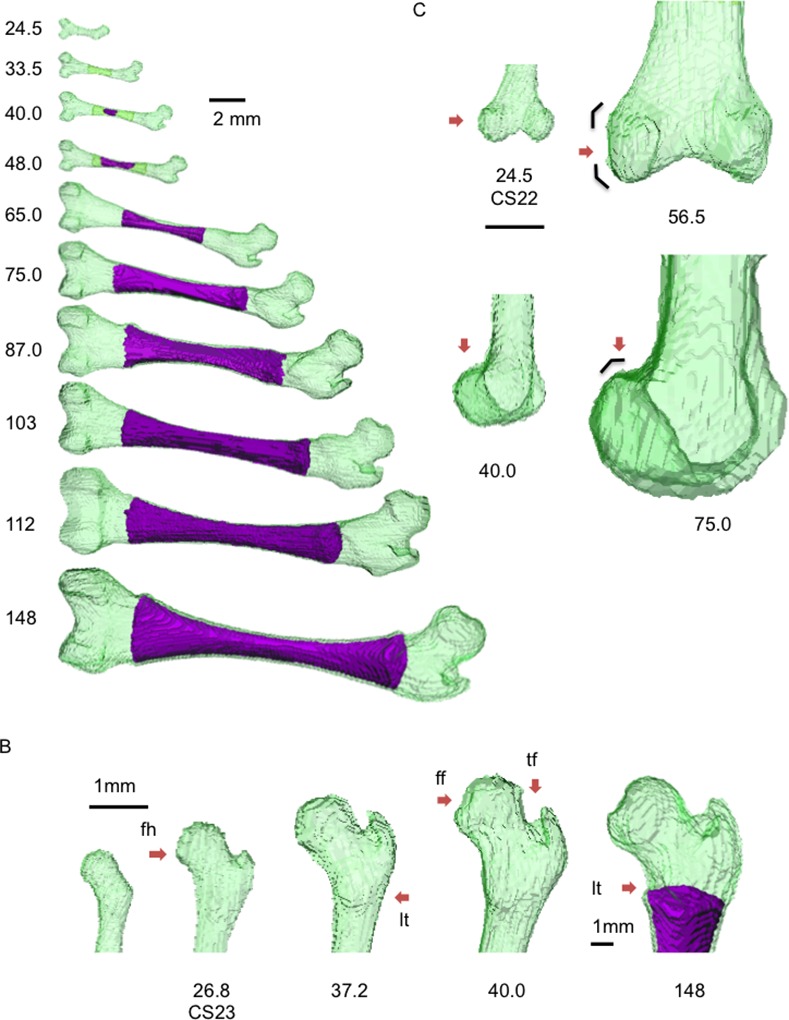
Representative 3-D reconstruction of the right femur from the embryonic period to the early fetal period. (A) Gross view, (B) proximal epiphysis (posterior view), (C) distal epiphysis. Numbers represent the crown–rump length (mm). Ossified region is indicated as purple. ff: femoral head fovea; fh: femoral head; lt: lesser trochanter; tf: trochanteric fossa. See also S7, S8 and S9 Movies.

No characteristic external change was detected at the diaphysis, whereas internal differentiation—namely, femoral ossification—was identified at the center of the diaphysis in fetuses with a CRL of 40.0 mm (red arrow in Figs 4 and 5A), which spread toward both epiphyses and reached the lesser trochanter in fetuses with a CRL of 148 mm (Figs 4 and 5A).

As previously mentioned, the femoral neck was constricted and the femoral head and greater trochanter were discernable by the end of the embryonic period (CS23 [CRL of 26.8 mm]) (Fig 5B). The lesser trochanter was detected in embryos with a CRL of 26.8 mm (CS23), which became sharp in fetuses with a CRL of 37.2 mm. The greater trochanter and trochanteric fossa became evident in fetuses with CRL between 33.5 and 40.0 mm. The femoral head fovea was identified in fetuses with a CRL of 40.0 mm, which became obvious in fetuses with CRL between 37.2 and 45.0 mm. No particular changes at the proximal epiphysis were noted in fetuses with a CRL of ≥45.0 mm.

The condyles were round and spherical in fetuses with a CRL of 24.5 mm (CS22). The lateral condyle appeared round in fetuses with a CRL of 48.0 mm, which changed to angular in the posterior view in fetuses with a CRL of 56.5 mm (Fig 5C). The upper portions of the condyles also became angular in the lateral view in fetuses with a CRL of 75.0 mm.

#### Femur length and OSL

**Fig 6:**
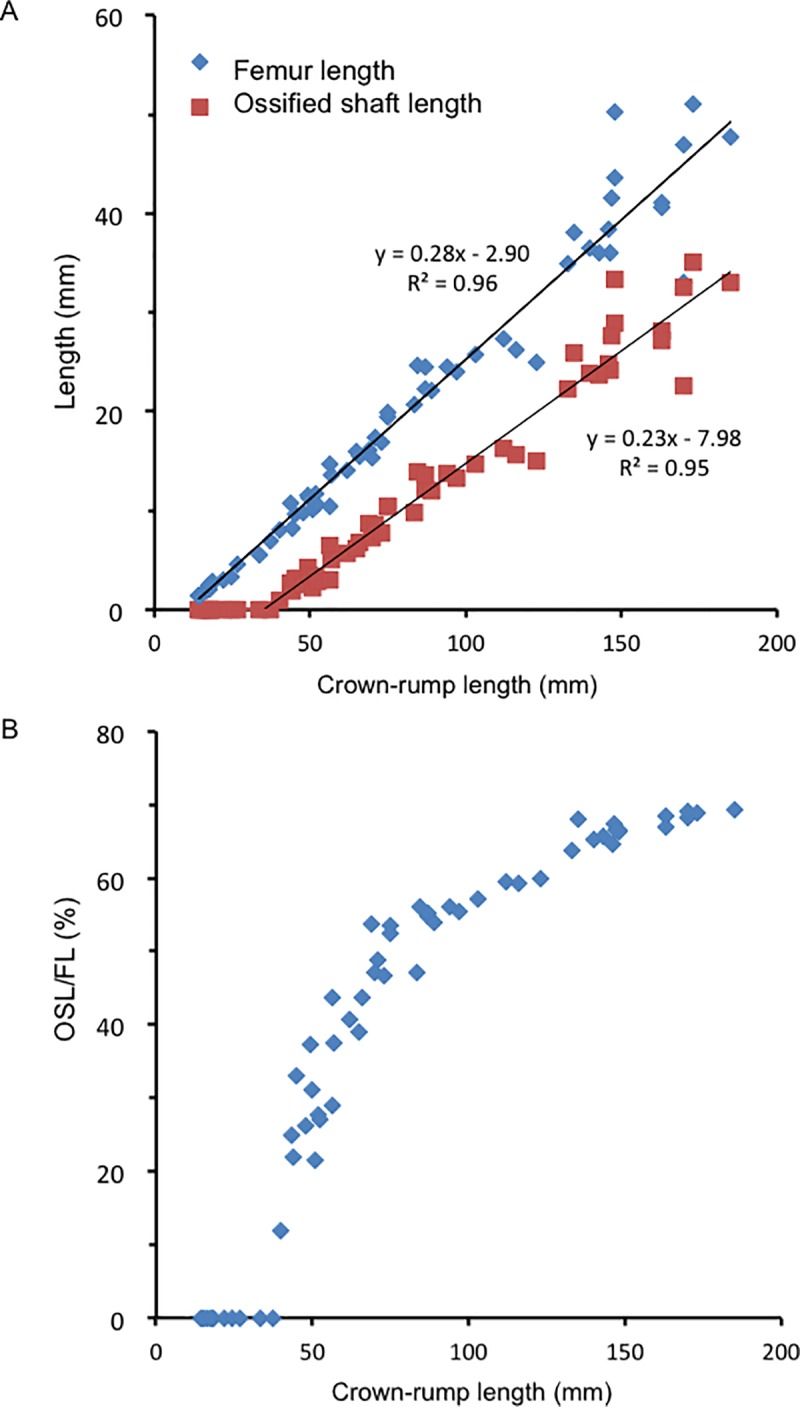
Growth and endochondral ossification of the femur according to crown–rump length (CRL). (A) Femur length and ossified shaft length (OSL) according to CRL. (B) OSL-to-femur length ratio according to CRL.

Femur length showed a strong positive correlation with CRL (R^2^ = 0.96) (Fig 6A). Ossification in the femoral shaft was detected in all specimens with a CRL of ≥40 mm. Similarly, OSL showed a strong positive correlation with CRL (R^2^ = 0.95) (Fig 6A). The OSL-to-femur length ratio rapidly increased by approximately 50% in fetuses with CRL between 40 and 75 mm (Fig 6B). The rate of increase became moderate in fetuses with a CRL of ≥75 mm, which reached to 70% in fetuses with a CRL of 185 mm.

#### Cartilage canal formation at the epiphysis

Incipient cartilage canal invasion at the proximal epiphysis was first observed on the surface of the femoral neck in fetuses with a CRL of 62 mm (OSL, 5.7 mm; Table 2). Cartilage canals at the trochanteric fossa were detected in fetuses with a CRL of 69 mm (OSL, 8.7 mm), which increased and attained the double-positive stage (++) of cartilage canal formation in all fetuses with a CRL of ≥75 mm (OSL, 9.8 mm). In contrast, cartilage canals at the femoral head fovea were observed in much larger fetuses and were detected for the first time in fetuses with a CRL of 84.5 mm (OSL, 13.8 mm).

**Table 2:** Evaluation of cartilage canals.

				Proximal epiphysis			Distal epiphysis	
ID	CRL (mm)	OSL (mm)	Fertilization* (days)	Neck	Trochanteric fossa	Fovea	Epicondyle	Intercondylar fossa
**52002**	**37.2**	**0.0**	**58**	**—**	**—**	**—**	**—**	**—**
**52730**	**40**	**1.0**	**62**	**—**	**—**	**—**	**—**	**—**
**33563**	**43.5**	**2.7**	**63**	**—**	**—**	**—**	**—**	**—**
**35726**	**44**	**1.8**	**63**	**—**	**—**	**—**	**—**	**—**
**52546**	**45**	**3.2**	**64**	**—**	**—**	**—**	**—**	**—**
**F2624**	**48**	**2.6**	**66**	**—**	**—**	**—**	**—**	**—**
**F2510**	**49.5**	**4.3**	**66**	**—**	**—**	**—**	**—**	**—**
**F2580**	**50**	**3.5**	**66**	**—**	**—**	**—**	**—**	**—**
**51262**	**51**	**2.2**	**67**	**—**	**—**	**—**	**—**	**—**
**51128**	**52**	**3.3**	**67**	**—**	**—**	**—**	**—**	**—**
**34365**	**52.3**	**2.9**	**67**	**—**	**—**	**—**	**—**	**—**
**52201**	**56.5**	**6.4**	**69**	**—**	**—**	**—**	**—**	**—**
**20799**	**56.5**	**3.0**	**69**	**—**	**—**	**—**	**—**	**—**
**F3049**	**57**	**5.1**	**70**	**—**	**—**	**—**	**—**	**—**
**51272**	**62**	**5.7**	**73**	**+**	**—**	**—**	**—**	**—**
**F2148**	**65**	**6.2**	**76**	**+**	**—**	**—**	**—**	**—**
**36175**	**66**	**6.8**	**76**	**—**	**—**	**—**	**—**	**—**
**F2214**	**69**	**8.7**	**77**	**+**	**+**	**—**	**—**	**—**
**37334**	**70**	**7.2**	**78**	**+**	**+**	**—**	**—**	**—**
**52248**	**71**	**8.5**	**78**	**(+)**	**(+)**	**—**	**—**	**—**
**36530**	**72.7**	**7.8**	**79**	**+**	**+**	**—**	**—**	**—**
**52559**	**75**	**10.4**	**80**	**++**	**++**	**±**	**+**	**—**
**51732**	**75**	**10.4**	**80**	**++**	**++**	**—**	**+**	**+**
**38641**	**83.5**	**9.8**	**85**	**++**	**++**	**—**	**—**	**+**
**F2949**	**84.5**	**13.8**	**85**	**++**	**++**	**+**	**+**	**+**
**50673**	**87**	**12.2**	**88**	**++**	**++**	**+**	**+**	**++**
**34192**	**87**	**13.6**	**88**	**++**	**++**	**+**	**++**	**++**
**53514**	**89**	**12.0**	**ND**	**++**	**++**	**+**	**++**	**++**
**F1519**	**94**	**13.7**	**ND**	**++**	**++**	**—**	**++**	**+**
**53520**	**97**	**13.3**	**ND**	**++**	**++**	**+**	**++**	**++**
**33996**	**103**	**14.7**	**ND**	**++**	**++**	**+**	**++**	**++**
**53209**	**112**	**16.3**	**ND**	**++**	**++**	**++**	**++**	**++**

Cartilage canal formation was evaluated by counting the number of cartilage canals and dividing them into three stages (-: 0, +: 1–4, ++: >5). Age indicate the gestational days after last menstration. Abbreviations: CRL, crown–rump length; OSL, ossified shaft length.

*: accorcing to ultrasound data of in vitro fertilizations that relate CRL and gestational age in fetuses (https://www.babymed.com/fetus-crown-rump-length-crl-measurements-ultrasound)

Cartilage canals at the distal epiphysis were detected simultaneously at the epicondyle and intercondylar fossa in fetuses with a CRL of 75 mm (OSL, 10.4 mm), which increased and attained the double-positive stage (++) of cartilage canal formation in almost all fetuses with a CRL of ≥87 mm. The data of the present study indicated that cartilage canal invasion occurred earlier at the proximal epiphysis than at the distal epiphysis.

### Femur modeling during the fetal period

#### Angle measurements

**Fig 7:**
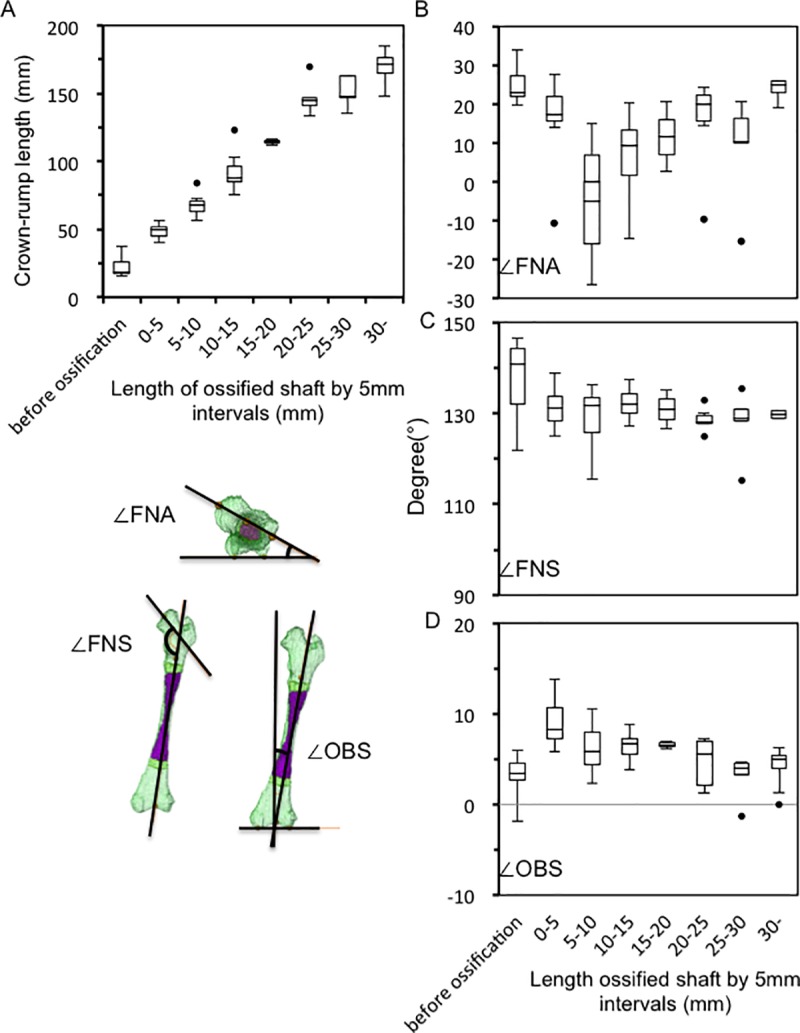
Femur modeling during the fetal period. (A) Distribution of crown–rump length (CRL) according to ossified shaft length (OSL). (B) Distribution of femoral neck anteversion (∠FNA) according to OSL. (C) Distribution of femoral neck shaft angle (∠FNS) according to OSL. (D) Distribution of obliquity of the shaft (∠OBS) according to OSL. In the box plots, the bars represent the sample range, the boxes represent the second and third quartiles, and the middle line represents the median.

Fetal samples were grouped into eight according to OSL with 5-mm intervals. CRL linearly increased according to OSL groups (Fig 7A). ∠FNA decreased from a median of 23.0° before ossification to a median of -4.8° in fetuses with an OSL of 5–10 mm (Fig 7B). The angle increased, reaching a median of 24.9° in fetuses with an OSL of >30 mm. The median ∠FNS was 140.8° before ossification (Fig 7C), and the distribution of median ∠FNS was relatively constant (a median of 130°) in all fetuses after ossification. Variation was prominent among fetuses before ossification (range: 121.9–146.7°). ∠OBS increased from 3.5° before ossification to a median of 9.1° in fetuses with an OSL of 0–5 mm (Fig 7D). The angle decreased to a median of 3.1° in fetuses with an OSL of 25–30 mm, which finally reached to a median of 4.4° in fetuses with an OSL of >30 mm. Bilateral differences in ∠FNA, ∠FNS, and ∠OBS, which were examined using Wilcoxon signed-rank test, were not observed in all fetuses (p = 0.98, 0.28, and 0.76 for ∠FNA, ∠FNS, and ∠OBS, respectively).

#### Procrustes analysis

**Fig 8:**
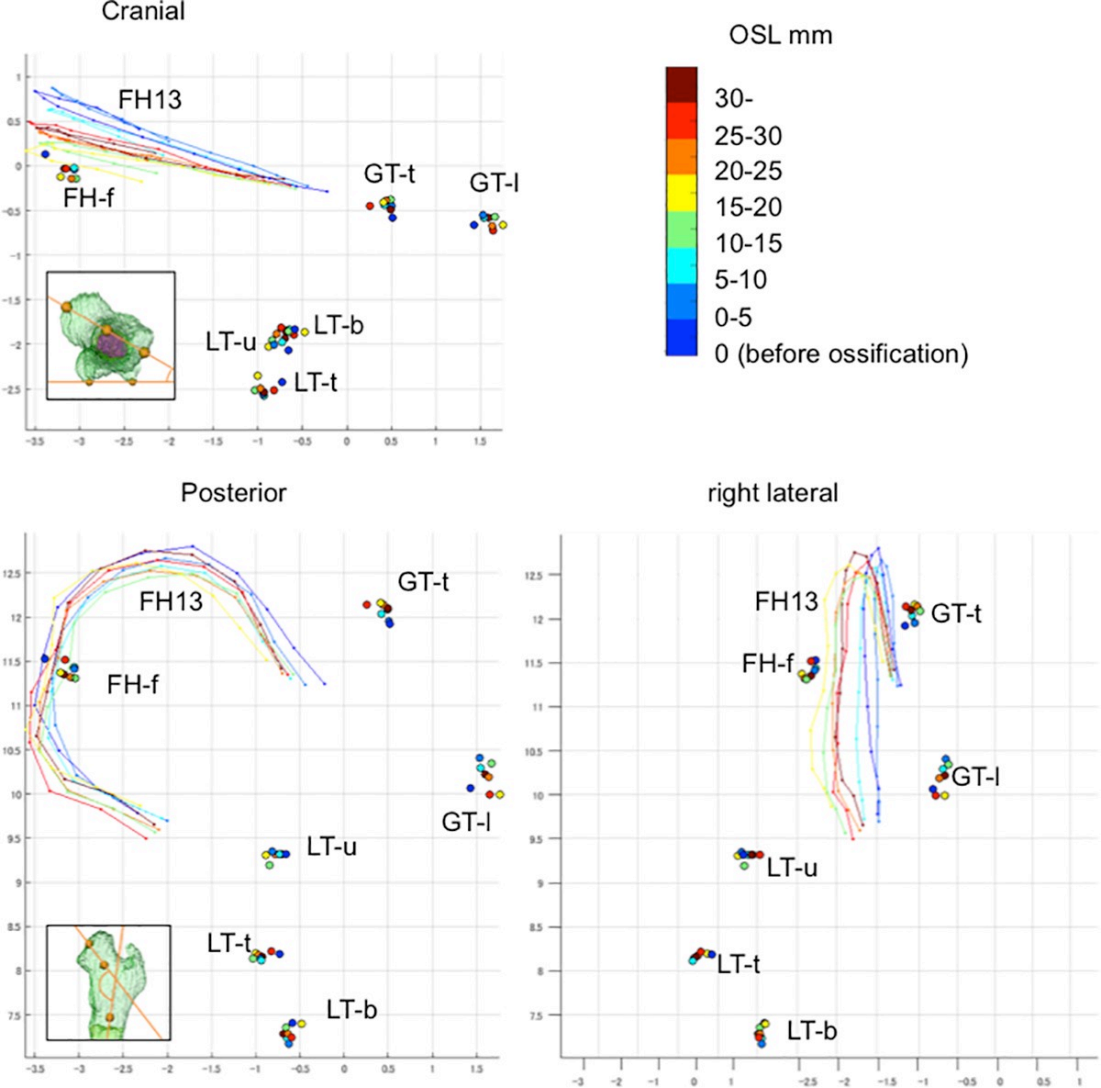
Reconstructed Procrustes shape coordinates for the proximal epiphysis. FH-f: center of the femoral head fovea; GT-l: most lateral point of the greater trochanter; GT-t: top of the greater trochanter; LT-b: bottom end of the lesser trochanter; LT-t: top of the lesser trochanter; LT-u: upper end of the lesser trochanter; FH13: 13 semi-landmarks from the upper end to the lower end of the femoral head along the plane passing through the midpoint of the femoral head, femoral neck, and greater trochanter.

Centroid sizes at both the proximal and distal epiphyses showed a strong positive correlation with OSL (R^2^ = 0.99 and 0.99) (S4 Fig). The Procrustes shape coordinates for the proximal epiphysis indicated that each landmark on the greater and lesser trochanters and femoral head fovea was located in the same position irrespective of OSL groups of fetuses (Fig 8). In comparison, semi-landmarks (FH13), which lined the femoral head, moved in accordance with the increase in OSL. Because FH13 evaluated the form and torsion of the femoral head, these movements might be correlated with the change in ∠FNA.

The Procrustes shape coordinates for the distal epiphysis indicated that each landmark was located in the same position irrespective of OSL groups of fetuses (S5 Fig). Semi-landmarks were located in different positions according to OSL groups, although no obvious regularity was noted.

## Discussion

Bardeen (1905) described that the chief features of the femur achieve the characteristics of the adult bone structure in the cartilaginous stage [10]. Images of the lower leg including the femur were provided in this previous study; unfortunately, the precise features could not be confirmed in these images. Our study clearly showed the femur before ossification in gross 3-D view and revealed the timeline for the development of the chief features of the femur. In particular, the greater and lesser trochanters and femoral head at the proximal epiphysis and the lateral and medial condyles at the distal epiphysis were detected by the end of the embryonic period (CS23). The fovea and trochanteric fossa were prominent during the early fetal period. The timeline for the embryonic landmarks seems to be genetically determined. However, the subsequent differentiation of anatomic landmarks likely involves the contributions of synchronized development of the surrounding joints, tendons, and muscles, as these landmarks are components for the attachment of muscle tendons and ligaments and are susceptible to movement-related changes on the joint surface.

The gross view of the cartilaginous femur in fetuses may be already similar to—but not identical to—that in adults. The structure of the cartilaginous femur may require modification. One opportunity for modification occurs at the time of initial endochondral ossification and shortly after that, which was detected as the change in angle measurements (∠FNA, ∠FNS, and ∠OBS) between the “before ossification” group and the “OSL 0–5 mm” group, as well as the large variances in the “OSL 0–5 mm” group. Angle measurements reflect the torsion and flexion of the femur but are unrelated to longitudinal growth.

The opportunity for modification may persist during the fetal period and even after birth. Semi-landmarks corresponding to the torsion of the femoral neck were moved and located in different positions during the fetal period. These movements might be correlated with ∠FNA, because both semi-landmarks and ∠FNA used the same plane as reference (i.e., the plane passing through the midpoint of the femoral head, femoral neck, and greater trochanter). The angles were various, particularly in samples at the time of ossification. Furthermore, negative anteversion was even observed. The change in anteversion during the fetal period was prominent compared with ∠FNS and ∠OBS in the present study [12]. Previous studies have reported variations in anteversion; specifically, the ∠FNA value ranged from -26° to 64° [12], from 17° to 45° [13], and from -14° to 41° [14]. Torsion of the lower leg including the femur (anteversion) was continuously observed during the fetal period and after birth [12,23,24]. Torsion of the femur may be prone to be affected by repetitive and persistent mechanical forces and intrauterine position [3]. Severe intrauterine compressive forces may result in congenital rotational limb deformities [25]. With respect to mechanical forces, muscle tensions and local forces exert rotary stress on the epiphysis, leading to the development of ∠FNA. Further studies should investigate the detailed mechanism concerning torsion of the femur.

Few shape changes except for the torsion mentioned above were observed in fetuses with an OSL of ≥10 mm. Procrustes analysis showed that all anatomical landmarks were located in the same position. Remodeling at the metaphysis and epiphysis during the growth of a long bone such as the femur is well known as the mechanism that maintains the shape [2]. For remodeling, osseous tissues have to be added and removed at the metaphysis and epiphysis of the developing long bone. Anatomical landmarks remained in the same relative position during subsequent endochondral ossification in the present study, indicating that the remodeling system during femur shaft growth in the longitudinal direction is elaborate.

In the present study, internal femoral changes in the chondrogenic stage and subsequent endochondral ossification were successfully detected by changing the signal intensity at the diaphysis and metaphysis. We could evaluate the correlation with histological and MRI findings using embryonic rat samples (S1 Supplementary Experiments). The data indicated that the histological findings corresponding to phase 4 according to Streeter’s classification showed low signal intensity compared with phases 1–3, whereas the histological findings corresponding to phase 5 showed high signal intensity (Fig 1) [22]. Considering that fluids such as blood and extracellular fluid collections exhibit high signal intensity on MRI, the difference in signal intensity between phases 4 and 5 might have resulted from the influx of blood flow and degeneration of cartilage cells at phase 5. The change in phase 4 and 5 regions could have followed; that is, phase 4 and 5 regions were first detected at the center of the diaphysis in fetuses with a CRL of 37.5 mm, which moved from the central part of the diaphysis to the metaphysis. Phase 4 and 5 regions became narrow, which might become the epiphyseal cartilage plate in larger fetuses.

Evaluation of fetal development using sonography is clinically performed mainly after the second trimester. OSL measurement in the femur for fetal growth assessment and pregnancy dating is accepted worldwide because femur length and OSL show a strong positive correlation with CRL [4,12]. Such strong correlation was also confirmed in the present study. In contrast, findings for morphological features in the femur have not yet been applied to the diagnosis of skeletal dysplasia. Currently, limited studies have reported the reference values for fetal limb bones at 9–16 weeks of gestation using transvaginal sonography [26,27]. Recent studies have indicated the possibility to detect various structural anomalies at 9–12 weeks of gestation by transvaginal sonography [28]. The results of the present study might aid in the application of morphological findings for the accurate clinical diagnosis of abnormal structure.

The present study has some limitations. First, we could not directly compare the histological findings for the femur and 7-T MR images acquired in the present condition using human fetal specimens because we are not allowed to destroy the human fetal specimens. Instead, the correlation with histological and MRI findings could be evaluated using embryonic rat samples. Second, our samples were stored in a medium containing formaldehyde for a long period. We speculate that the differentiation and maturation of the diaphysis may affect the change in signal intensity in that region. However, it cannot be excluded that conditions such as long-term fixation in each sample may alter the differences in signal intensity. The possibility that morphology and several morphometric data were affected as a result of fixation or the process of termination cannot be excluded. Third, our samples, which were obtained by artificial abortion, were recognized as externally normal; nonetheless, it is not guaranteed that all samples show normal and standard development. Fourth, we employed several kinds of image acquisition methods based on specimen resolution and volume. The data provided by images acquired using PCX-CT and MRI are not identical, although the approximate morphology is comparable. Finally, the synchronized development of the surrounding muscles and joint formations was not analyzed together with femur formation, because the image resolution in the present study was not so high to precisely detect the attachments of the muscle tendons and joint ligaments.

In conclusion, the present study provided a useful standard for the morphogenesis of the femur, which could serve as a basis to better understand femur development and could aid in differentiating normal and abnormal development.

## Supporting information

S1 Supplementary Experiments Comparison of histological findings and MR images using rat femurs. (DOCX)

S1 Movie 3-D reconstruction of the femur at CS18. (AVI)

S2 Movie 3-D reconstruction of the femur at CS19. (AVI)

S3 Movie 3-D reconstruction of the femur at CS20. (AVI)

S4 Movie 3-D reconstruction of the femur at CS21. (AVI)

S5 Movie 3-D reconstruction of the femur at CS22. (AVI)

S6 Movie 3-D reconstruction of the femur at CS23. (AVI)

S7 Movie 3-D reconstruction of the femur during the early fetal period (CRL, 40 mm). (AVI)

S8 Movie 3-D reconstruction of the femur at CS23 during the fetal period (CRL, 75 mm). (AVI)

S9 Movie 3-D reconstruction of the femur during the fetal period (CRL, 148 mm). (AVI)

S1 Fig Comparison of MRI data and histological findings using embryonic rat samples (embryonic day 18). (A) Longitudinal sections of the left femur on embryonic day 18 with histological staining (hematoxylin and eosin staining and safranin O staining; ×40); 7-T MR images are shown. Red arrow: phase 4; yellow arrow: phase 5; asterisk: phase OS.
(B) High magnification showing endochondral ossification. Histology belongs to phases 3–5 and phase OS. *: perforation of the periosteal collar. 3: phase 3; 4: phase 4; 5: phase 5; OS: phase OS.
(C) 7-T MR images of the femur from human fetuses (CRL, 40 mm; ID: 52730), which have similar findings. Red arrow: phase 4; yellow arrow: phase 5; asterisk: phase OS.
(TIF)

S2 Fig Comparison of MRI data and histological findings using embryonic rat samples (embryonic day 19). (A) Longitudinal sections of the left femur on embryonic day 19 with histological staining (hematoxylin and eosin staining and safranin O staining; ×20); 7-T MR images are shown. Red arrow: phase 4; yellow arrow: phase 5; asterisk: phase OS.
(B) High magnification showing endochondral ossification. Histology belongs to phases 3–5 and phase OS. c: capillary; po: periosteal collar; os: osteoid. 3: phase 3; 4: phase 4; 5: phase 5.
(C) 7-T MR images of the femur from human fetuses (CRL, 56.5 mm; ID: 52201), which have similar findings. Red arrow: phase 4; yellow arrow: phase 5.
(TIF)

S3 Fig Comparison of MRI data and histological findings using embryonic rat samples (embryonic day 20). (A) Longitudinal sections of the left femur on embryonic day 20 with histological staining (hematoxylin and eosin staining and safranin O staining; ×20); 7-T MR images are shown. Red arrow: phase 4; yellow arrow: phase 5; asterisk: phase OS.
(B) High magnification showing endochondral ossification. Histology belongs to phases 3–5 and phase OS. 3: Phase 3; 4: phase 4; 5: phase 5; OS: phase OS.
(C) 7-T MR images of the femur from human fetuses (CRL, 75.0 mm; ID: 52559), which have similar findings. Red arrow: phase 4; yellow arrow: phase 5.
(TIF)

S4 Fig Procrustes shape coordinates. (A) Centroid size of the proximal epiphysis according to ossified shaft length (OSL). (B) Centroid size of the distal epiphysis according to OSL.
(TIF)

S5 Fig Reconstructed Procrustes shape coordinates of the distal epiphysis. IF: center of the intercondylar fossa; LC-B: bottom point of the lateral condyle; LC-p: most posterior point of the lateral condyle; LE: most lateral point of the lateral epicondyle; MC-p: most posterior point of the medial condyle; MC-b: bottom point of the medial condyle; ME: most lateral point of the medial epicondyle; LC13: 13 semi-landmarks along the roundness of the lateral condyle from the upper end to the opposite side; MC13: 13 semi-landmarks along the roundness of the medial condyle from the upper end to the opposite side.
(TIF)
