# Supplementary material for: Morphogenesis of the femur at different stages of normal human development
Source: PLoS One. 2019 Aug 23;14(8):e0221569. doi: 10.1371/journal.pone.0221569 (PMC6707600; doi:10.1371/journal.pone.0221569)
Supplement: S1 Supplementary Experiments — (DOCX) [file pone.0221569.s001.docx]

**Supplementary experiments**

**Comparison of histological findings and MR images using rat femurs**

To compare histological findings and 7-T MR images used in the present study, the lower limbs of rats were subjected initially to 7-T MR image acquisition and subsequently to histological sectioning. Pregnant Wistar rats were purchased from Shimizu Laboratory Supplies Co., Ltd. (Kyoto, Japan). On embryonic days (ED) 18, 19, and 20, fetuses were excised from each pregnant rat (n=3), and the lower limbs were used for analysis. Specimens were dehydrated with ethanol and xylene, embedded in paraffin, and subsequently cut into 8-µm-thick longitudinal sections. All sections were stained with hematoxylin and eosin and with safranin O/fast green/hematoxylin.

Endochondral ossification was evaluated histologically. Phases 1–5 were defined according to Streeter’s classification [1]. Briefly, histological findings for the five successive phases in cartilage tissues were as follows:

Phase 1: Cells emerging from the skeletal blastema become slightly larger; active proliferation.

Phase 2: Slender cells with a trace of vacuolization; active proliferation.

Phase 3: Cells larger by threefold or more than those at phase 2; cuboidal cells with numerous vacuoles.

Phase 4: Cartilage cells of maximum size; cytoplasm shows extreme vacuolization; intercellular substance has become more opaque, with an increased amount of coagulable material, and resembles a honeycomb.

Phase 5: Cartilage cells disintegrated in varying degrees; the intercellular honeycomb is left with vacated compartments to some extent.

Phase OS was provided when histological findings exceeded the criteria for phase 5. Experiments with animals were approved by the institutional animal research committee and were performed in accordance with the Guidelines for Animal Experiments of Kyoto University (permit number: 14038). Care of animals conformed to the Kyoto University guidelines.

Cartilage cells in the femur of rats on ED18 were at the center of the diaphysis (phase OS), as primary trabeculae, capillary formation, and osteoid were observed (S7 Fig). Adjacent to the region at phase OS, the regions at phases 5, 4, 3, 2, and 1 were identified from the diaphysis to the epiphysis in this order. Histology revealed greatly hypertrophied and fragmented chondrocytes at phase 5. Both ends of fragmented chondrocytes were hypertrophied (phase 4). Both epiphyses (phases 1–3) and hypertrophied chondrocytes with matrix (phase 4) stained red with safranin O, indicating that the cartilage matrix was present in these regions. In contrast, tissues in the middle of the shaft at phase 5 and phase OS did not stain red with safranin O. On 7-T MR images, the center of the shaft in the femur on ED18 showed high signal intensity, which corresponded to phase 5 and phase OS. In regions adjacent to the high signal intensity, low signal intensity bands were observed, which might correspond to phase 4.

The phase OS region expanded in the diaphysis on ED19, whereas the phase 4 and 5 regions decreased compared with those observed in the femur on ED18 (S8 Fig). The phase OS region that expanded in the diaphysis showed a mixture of low and high signal intensity (netlike pattern) on MRI, which corresponded to the trabecular bone matrix. The phase 5 region adjacent to the region at phase OS had high signal intensity. In regions adjacent to the high signal intensity, low signal intensity bands were observed, which might correspond to phase 4.

The trabecular network expanded and cancellous bones formed at the center of the shaft in the femur on ED20 (S9 Fig). Phase 4 and 5 regions appeared similar to a thin layer, which might become the epiphyseal cartilage plate (growth plate or physis). On 7-T MR images, the center of the shaft in the femur showed low signal intensity. The metaphysis had both sharp and thin high signal intensity band (phase 5) along with ambiguous low signal intensity (phase 4).

The periosteal collar, which differentiates into the cortical bone, was histologically observed in samples on ED18–20. All borders of the femur were recognized as regions with high signal intensity, and the periosteal color was not discernable from the border of the epiphysis on MRI.

MR images of femurs from embryonic rats and human fetuses were subsequently compared (S7, S8, and S9 Figs). Longitudinal sections of the right femur from rats on ED18–20 and those from fetuses with CRLs of 40 mm, 56.5 mm, and 75.0 mm had similar findings on MR images.

These findings indicated that the femur during endochondral ossification can be divided into five successive phases according to Streeter’s classification and phase OS using hematoxylin and eosin-stained sections in embryonic rats. Moreover, phases 1–5 and phase OS can be discernable on MR images. Ossified regions (phase OS) exhibited low signal intensity or a mixture of low and high signal intensity, whereas phase 5 regions showed high signal intensity. Phase 4 regions showed low signal intensity; however, the border at the epiphysis from phases 1 to 3 was occasionally ambiguous. Such MRI findings may be applicable to samples from human fetuses.

**Reference**

[1] Streeter GL. Developmental horizons in human embryos (fourth issue). A review of the histogenesis of cartilage and bone. Contrib Embryol. 1949;33: 149-168.
