# Supplementary figures and images for: Morphogenesis of the femur at different stages of normal human development
Source: PLoS One. 2019 Aug 23;14(8):e0221569. doi: 10.1371/journal.pone.0221569 (PMC6707600; doi:10.1371/journal.pone.0221569)

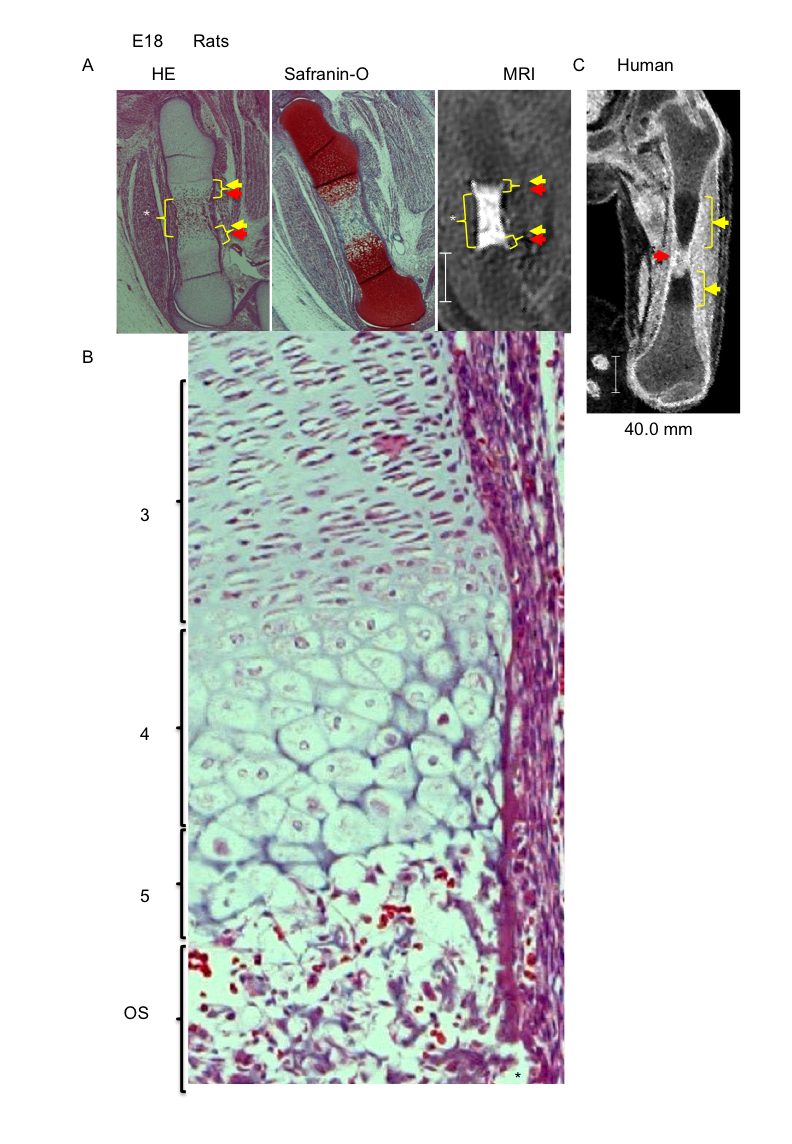

Supplement: S1 Fig — (A) Longitudinal sections of the left femur on embryonic day 18 with histological staining (hematoxylin and eosin staining and safranin O staining; ×40); 7-T MR images are shown. Red arrow: phase 4; yellow arrow: phase 5; asterisk: phase OS. (B) High magnification showing endochondral ossification. Histology belongs to phases 3–5 and phase OS. *: perforation of the periosteal collar. 3: phase 3; 4: phase 4; 5: phase 5; OS: phase OS. (C) 7-T MR images of the femur from human fetuses (CRL, 40 mm; ID: 52730), which have similar findings. Red arrow: phase 4; yellow arrow: phase 5; asterisk: phase OS. (TIF) [file pone.0221569.s011.tif]

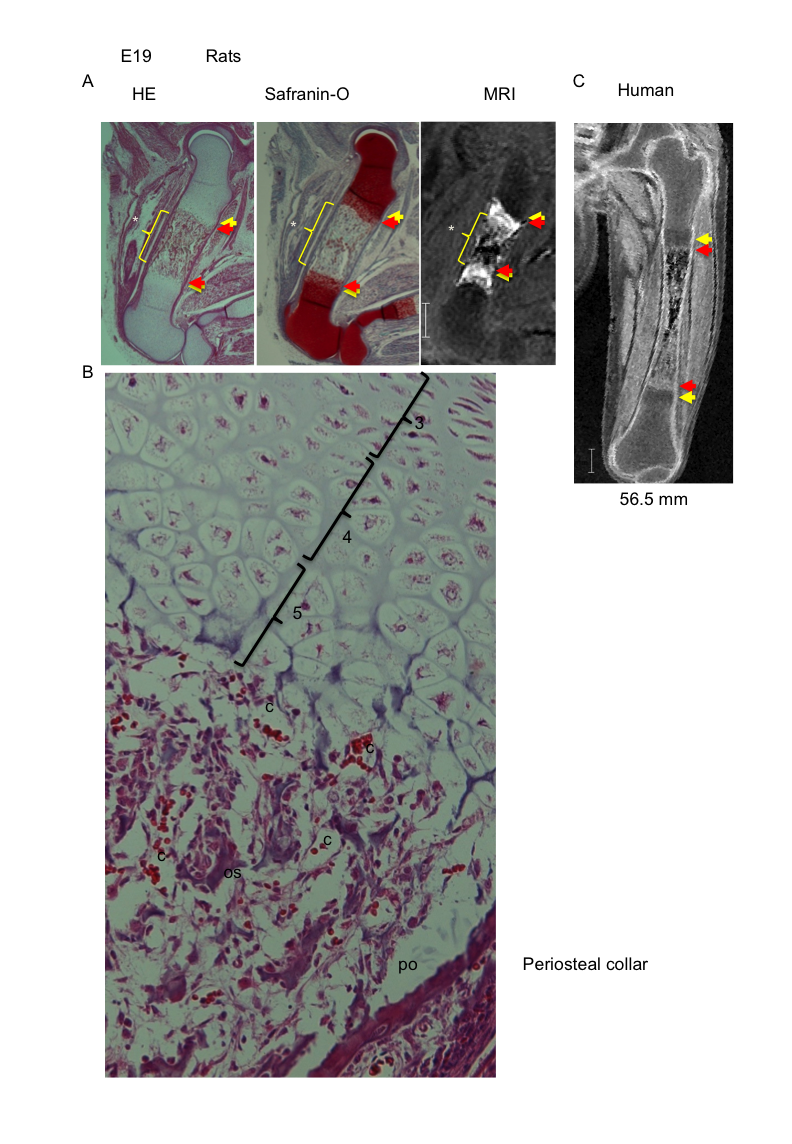

Supplement: S2 Fig — (A) Longitudinal sections of the left femur on embryonic day 19 with histological staining (hematoxylin and eosin staining and safranin O staining; ×20); 7-T MR images are shown. Red arrow: phase 4; yellow arrow: phase 5; asterisk: phase OS. (B) High magnification showing endochondral ossification. Histology belongs to phases 3–5 and phase OS. c: capillary; po: periosteal collar; os: osteoid. 3: phase 3; 4: phase 4; 5: phase 5. (C) 7-T MR images of the femur from human fetuses (CRL, 56.5 mm; ID: 52201), which have similar findings. Red arrow: phase 4; yellow arrow: phase 5. (TIF) [file pone.0221569.s012.tif]

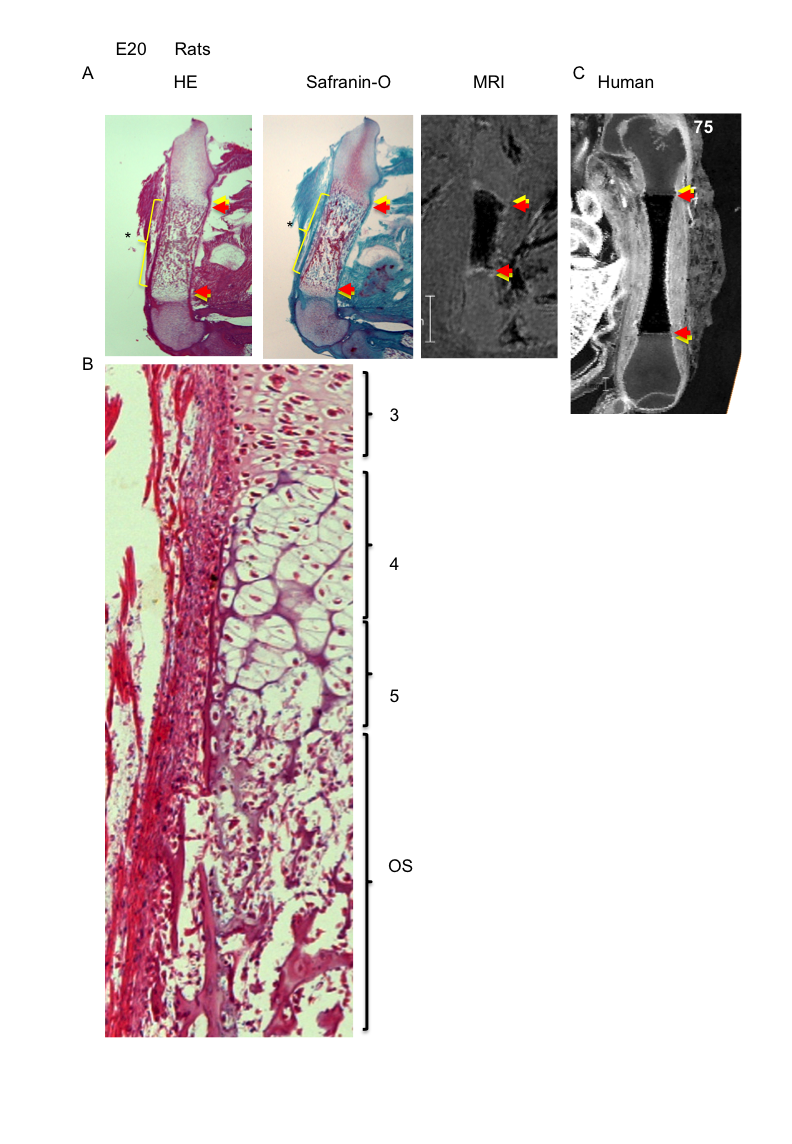

Supplement: S3 Fig — (A) Longitudinal sections of the left femur on embryonic day 20 with histological staining (hematoxylin and eosin staining and safranin O staining; ×20); 7-T MR images are shown. Red arrow: phase 4; yellow arrow: phase 5; asterisk: phase OS. (B) High magnification showing endochondral ossification. Histology belongs to phases 3–5 and phase OS. 3: Phase 3; 4: phase 4; 5: phase 5; OS: phase OS. (C) 7-T MR images of the femur from human fetuses (CRL, 75.0 mm; ID: 52559), which have similar findings. Red arrow: phase 4; yellow arrow: phase 5. (TIF) [file pone.0221569.s013.tif]

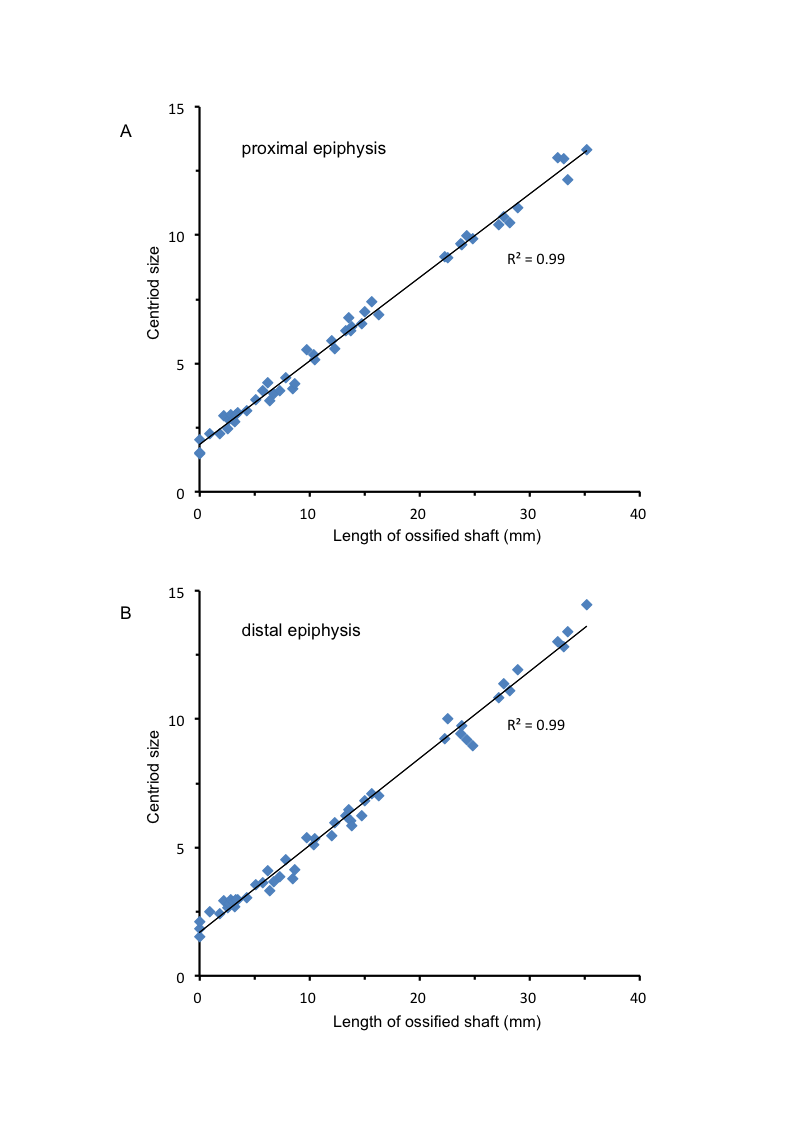

Supplement: S4 Fig — (A) Centroid size of the proximal epiphysis according to ossified shaft length (OSL). (B) Centroid size of the distal epiphysis according to OSL. (TIF) [file pone.0221569.s014.tif]

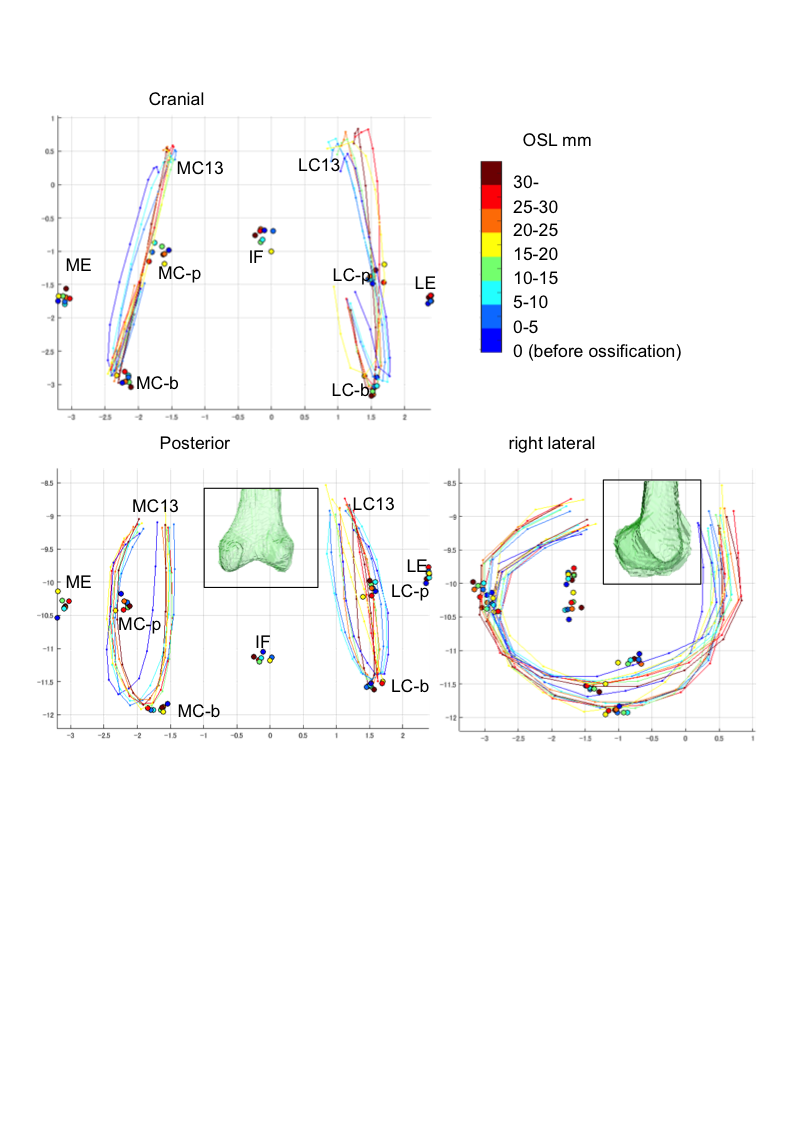

Supplement: S5 Fig — IF: center of the intercondylar fossa; LC-B: bottom point of the lateral condyle; LC-p: most posterior point of the lateral condyle; LE: most lateral point of the lateral epicondyle; MC-p: most posterior point of the medial condyle; MC-b: bottom point of the medial condyle; ME: most lateral point of the medial epicondyle; LC13: 13 semi-landmarks along the roundness of the lateral condyle from the upper end to the opposite side; MC13: 13 semi-landmarks along the roundness of the medial condyle from the upper end to the opposite side. (TIF) [file pone.0221569.s015.tif]
